# Error‐related brain activity associated with obsessive–compulsive symptoms in youth

**DOI:** 10.1002/brb3.2941

**Published:** 2023-03-14

**Authors:** Hannah Becker, Yanni Liu, Gregory L. Hanna, Emily Bilek, Stefanie Russman Block, Jillian E. Hardee, Mary M. Heitzeg, David Pagliaccio, Rachel Marsh, Kate D. Fitzgerald

**Affiliations:** ^1^ Department of Psychology, University of Michigan Ann Arbor Michigan USA; ^2^ Department of Psychiatry University of Michigan Ann Arbor Michigan USA; ^3^ Addiction Research Center University of Michigan Ann Arbor Michigan USA; ^4^ New York State Psychiatric Institute Columbia University New York New York USA

**Keywords:** community sample, dimensional approach, dorsal anterior cingulate cortex, error processing, obsessive–compulsive symptoms, youth

## Abstract

**Background:**

Subclinical obsessive–compulsive symptoms (OCS) are common in children, and increase risk for later onset of obsessive–compulsive disorder (OCD). In pediatric patients with OCD, neuroimaging research implicates altered neural mechanisms for error‐processing, but whether abnormal brain response occurs with subclinical OCS remains poorly understood.

**Methods:**

Using functional magnetic resonance imaging (fMRI), 113 youth (8–18 years; 45 female) from a community sample were scanned during an error‐eliciting Go/No‐Go task. OCS were assessed dimensionally using the obsessive–compulsive subscale of the Child Behavior Checklist. The association between OCS scores and error‐related brain activity was examined at the whole‐brain level.

**Results:**

Lower OCS scores associated with stronger response to errors in dorsal anterior cingulate cortex (dACC), caudate, putamen, thalamus, and occipital cortex. Additionally, lower OCS related to higher capacity for inhibitory control, as indexed by greater accuracy on No‐Go trials during fMRI scanning. The relationship between lower OCS and better accuracy on No‐Go trials was mediated by greater error‐related dACC activity.

**Conclusions:**

The inverse relationship between OCS and error‐related activity in the dACC and extended cortical–striatal–thalamic circuitry may index an adaptive process by which subclinical OCS are minimized in youth. Further, these results identify an observable pattern of brain activity that tracks with subclinical OCS severity. Understanding the link between neural networks for error processing and the normal to abnormal range of OCS may pave the way for brain‐based strategies to identify children who are more likely to develop OCD and enable the targeting of preventive strategies to reduce risk.

## Graphical abstract

Graphical Abstract: This fMRI study examines the association of error‐related brain activity and dimensionally measured obsessive–compulsive symptoms (OCS) in a community sample of youth. Results showed that lower OCS was associated with greater error‐related activity of the dorsal anterior cingulate cortex (dACC), caudate, putamen and thalamus, as well as better task performance. This finding provides new information about error‐related brain activity in youth with OCS and sheds light on neural markers that might be relevant for the later development of OCD pathology.

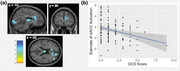

## 1 INTRODUCTION

Obsessive–compulsive disorder (OCD) is a highly impairing disorder where patients experience obsessive thinking patterns and compulsive urges or behaviors; it is estimated that about 1%–2% of the population experiences clinical OCD (Ruscio et al., 2010). However, these symptoms also exist at the subclinical level, carrying risk for the development of clinical OCD (Fullana et al., 2010). In fact, subclinical obsessive–compulsive symptoms (OCS) affect up to 40% of children (Barzilay, 2019; Fullana et al., 2009; Valleni‐basile et al., 1994), cause functional impairment, and increase risk for future OCD (Barzilay, 2019; Weeland et al., 2021).

The association of OCS with a later development of OCD implicates OCS as a potential target for intervention, yet strategies for distinguishing children, who will “grow out” of symptoms from those who will develop OCD, remain to be established. Further, many studies evaluating the neurobiological correlates of OCD have focused on group differences between patients with OCD and healthy individuals, potentially obscuring unique associations between brain and OCS that are observable when evaluated in a wider population of youth with a continuous range of symptoms. Establishing quantifiable neural markers associated with youth OCS in a cross‐sectional sample is a first step toward mechanism‐based identification of youth most at risk for developing OCD and targeted prevention (Casey et al., 2014). For instance, recent cross‐sectional studies have used fMRI to assess how resting‐state functional connectivity associated with OCS in youth, with the goal of detecting a neuroimaging marker that tracks subclinical symptoms and could be tested in future, longitudinal work to identify children most at risk for later OCD (Alexander‐Bloch et al., 2022; Pagliaccio et al., 2021). These resting‐state studies suggest patterns of connectivity between cortical–striatal–thalamic–cortical circuitry (CSTC; Suñol et al., 2021) and task control network regions (Agam et al., 2014; Alexander‐Bloch et al., 2022; Pagliaccio et al., 2021) associate with OCS in youth and have potential as risk predictors. However, error‐related, *task‐based* fMRI, as evaluated here, may provide a specific advantage in identifying neurobiology associated with OCS, as error‐processing deficits are at the core of OCD pathology (Abramowitz et al., 2006).

Error‐related brain activity has been studied using both electroencephalography (EEG) and functional magnetic resonance imaging (fMRI) approaches and is typically linked to the dorsal anterior cingulate cortex (dACC) and surrounding posterior medial frontal cortex (pMFC; Overbye et al., 2019) among other, more widely distributed regions (e.g., insula; Iannaccone et al., 2015). At the core of neural networks for error processing, the pMFC and its subregions have been implicated in error‐related functions of performance monitoring, error‐detection, and interference processing in youth and adults (Fitzgerald, Perkins, et al., 2010; Ridderinkhof et al., 2004). Engagement of the pMFC enables individuals to perform goal‐directed behaviors and make associated, necessary adjustments to behavior.

EEG studies in pediatric and adult patients with OCD show an abnormally increased amplitude of the error‐related negativity (ERN; Moser et al., 2013, but see also Ip et al., 2019), an event‐related potential that follows an error and localizes to midline frontal cortex. Moreover, fMRI has been used to assess error‐processing function in OCD. Pooling data across fMRI studies of pediatric and adult OCD, meta‐analysis shows greater error‐related brain activity in patients as compared to controls, particularly in dACC and adjacent areas of the pMFC (Norman et al., 2019). Greater pMFC response to errors in OCD may reflect affective hypersensitivity to perceived errors that could drive compulsive attempts at correction (Fitzgerald et al., 2005; Olvet & Hajcak, 2008). Alternatively, greater error‐signaling may reflect an adaptive response, engaging neural networks for task control to override habitual behaviors and decrease compulsive ritualizing (Endrass & Ullsperger, 2014; Fitzgerald, Stern, et al., 2010).

Despite the superior spatial resolution of fMRI for assessing the distributed brain networks implicated in error‐processing and OCD (e.g. CSTC), there have been a disproportionately small number of studies assessing error processing with fMRI as compared to EEG, and none that have evaluated the association between error‐related activity and OCS at the subclinical level in youth. The motivation for the use of fMRI in the current study is to elucidate the association between brain response to errors in this youth age range, with the greater spatial precision of fMRI, and with increased power afforded by a comparatively large sample. To test pMFC‐based error signaling as a marker of subclinical OCS, we examined the relationship between error‐related brain activity and OCS, considered dimensionally, in a community sample of youth. Errors were elicited during fMRI scanning, using a well‐validated Go/No‐Go task that has been previously found to engage a network of error‐related regions, including the pMFC (Overbye et al., 2019; Steele et al., 2014). Given findings of greater pMFC‐based error‐processing activity in OCD when categorically compared to controls, one prediction is that more subclinical OCS would associate with greater pMFC response to errors. Conversely, greater response to errors may play an adaptive role, supporting less severe illness in pediatric OCD (Fitzgerald, Perkins, et al., 2010). Thus, the current analyses will allow for an empirical examination of these two competing hypotheses, using fMRI.

## 2 METHODS AND MATERIALS

### 2.1 Participants

Participants were drawn from the neuroimaging sub‐study of the Michigan Longitudinal Study (MLS; Zucker et al., 1996). The MLS is a multigenerational, prospective study oversampled for families affected by alcohol use disorders, thus yielding a sample of youth at risk for a broad range of psychopathology including OCS (Nurnberger et al., 2004). Inclusion criteria (see the Supporting Information section) were met by 113 individuals (68 male; 8.2–18.0 years [M = 13.14]) from the MLS neuroimaging sub‐study. Written informed consent from parents and assent from youth participants were obtained in writing, as approved by the University of Michigan Medical School Institutional Review Board.

### 2.2 Clinical assessment

The computerized Diagnostic Interview Schedule for Children (DISC; Shaffer et al., 2004) was completed by the youth's primary caregiver and used to assign current psychiatric diagnoses (Table 1). OCS were measured dimensionally with the OCS subscale of the Child Behavior Checklist (CBCL) (Achenbach, 1999; CBCL‐OCS) that sums four items from the CBCL‐Anxious/Depressed and four items from the CBCL‐Thought Problems syndrome scales (refer to Table S1 for specific items; Achenbach, 1999; Hudziak et al., 2006). Items are rated as 0 (“absent”), 1 (“occurs sometimes”), or 2 (“occurs often”), allowing for a continuous range of OCS scores from 0 to 16. CBCL‐OCS scores greater than 4 reflect high risk for developing OCD (Saad et al., 2017).

**TABLE 1:** Demographic and psychometric variables

**Age**: mean (SD)	13.1 (2.96)
**Sex**: male/female **Race**: American Indian or Alaska Native Asian Biracial Black or African American Native Hawaiian or other Pacific Islander White **Ethnicity**: Hispanic/non‐Hispanic	68/45 0 0 13 15 0 85 9/104
**DSM‐IV Past Month Diagnosis** (count):^a^	
Attention‐deficit/hyperactivity disorder	11
Anxiety disorder^b^	4
Depressive disorder^c^	3
Oppositional defiant disorder	10
**CBCL‐OCS** ^d^ **score** (count):	
0	38
1	31
2	19
3	10
4	8
5	5
6	1
9	1

^a^ Includes youth with comorbid diagnoses. Eight individuals also endorsed excessive alcohol use, though none met criteria for alcohol dependence.

^b^ Includes diagnoses of generalized anxiety disorder, panic disorder, and social anxiety disorder.

^c^ Depressive disorder: major depressive disorder and/or dysthymic disorder.

^d^ CBCL‐OCS, Child Behavior Checklist obsessive–compulsive subscale.

### 2.3 Error‐eliciting Go/No‐Go task

An event‐related Go/No‐Go task was used to elicit commission errors (failing to withhold a button press on a No‐Go trial) during fMRI, as in a previous work (Hardee et al., 2014). “Go” trials (∼75% of trials) required participants to press a button in response to letter target stimuli (all letters other than “X”). “No‐Go” trials (∼25% of trials) required the withholding of response to a nontarget stimulus (the letter “X”). On each trial, a letter stimulus was presented for 500 ms, with a 3500‐ms interval between stimuli during which participants viewed a black screen with a white fixation cross. Participants completed five 3.5‐min runs that each contained 49 trials (245 total trials; 60 No‐Go trials across all five runs). Prior to MRI scanning, all participants practiced one run (49 trials).

### 2.4 fMRI acquisition

Whole‐brain blood oxygen level‐dependent (BOLD) images and a high resolution anatomical scan were acquired on a 3.0‐T General Electric Signa scanner. Parameters of scan acquisition are included in the “Supplemental Methods” in the Supporting Information section.

### 2.5 Data analysis

#### 2.5.1 Behavioral

In‐scanner task performance was measured based on accuracy (percentage correct) on Go and No‐Go trials and mean RT on Go trials. Pearson correlations were calculated between CBCL‐OCS, task accuracy, RT, head motion (framewise displacement [FD]; Power et al., 2012), and age.

#### 2.5.2 fMRI analysis

Preprocessing of the neuroimaging data, including motion correction, slice timing correction, normalization, and smoothing, were done using in‐house pipelines (see “Supplemental Methods” in the Supporting Information section for additional information). First‐level analyses for each participant were conducted using a general linear model in SPM8, consistent with prior work (Hardee et al., 2014). Regressors for failed No‐Go trials (commission errors), correct No‐Go trials, and Go trials were convolved with the hemodynamic response function (4000‐ms event duration). Motion parameters and white matter signal intensity (derived from an anatomical mask) were also included as regressors, thus removing task‐unrelated noise. Second‐level group analyses examined error‐related activity (commission errors only), defined by the linear contrast of failed No‐Go versus correct No‐Go trials, as in prior studies (Hardee et al., 2014). Correct response inhibition was also examined, defined by the linear contrast of correct No‐Go versus correct Go trials. Contrasts for primary and secondary whole‐brain analyses were displayed at a peak (voxel level) threshold of *p* < .005, uncorrected, and clusters were considered significant at *p* < .05 (cluster level), corrected for false discovery rate (FDR) across the whole brain.

*Primary analysis: Main effect of OCS on error‐processing activity* . CBCL‐OCS scores were regressed on error‐related activity at the whole brain level, with regressors covarying for effects of age, mean FD, and task performance (No‐Go trial accuracy; Luna et al., 2010; Padmanabhan et al., 2011). Analyses, including these regressors, were designed to isolate the effects of OCS from confounding variables that can affect error processing (Moser et al., 2013; Overbye et al., 2019; Torpey et al., 2013) and were considered primary. Additional regression analyses were run to test this effect without the task performance covariate and also with a parental alcohol use disorder covariate (see “Supplemental Results” in the Supporting Information section). To compare with prior literature (Norman et al., 2019), whole‐brain analyses were conducted to also test the effect of CBCL‐OCS on brain activation during correct response inhibition. Assumptions for multiple linear regression were checked for three separate regressions of the same predictors on activity in the dACC, putamen, and occipital cortex, during task.

*Secondary analyses: Specificity of OCS effect on error‐processing activity*. Given that the CBCL‐OCS subscale overlaps with items from the CBCL‐Anxious/Depressed and CBCL‐Thought Problems subscales (Hudziak et al., 2006), secondary whole‐brain analyses were conducted to test the effect of scores for these subscales on activation to errors and response inhibition (see “Supplementary Results” in the Supporting Information section), covarying age, FD, and No‐Go accuracy. Details about additional tests of specificity can be found in the Supporting Information section.

## 3 RESULTS

### 3.1 Demographic and clinical characteristics

CBCL‐OCS scores were positively skewed and ranged from 0 to 9 (Table 1), consistent with prior work in community samples (Barzilay, 2019) and reflecting a range of OCS severity. Among the 113 youth, 7 participants (6.2%) had CBCL‐OCS scores above the threshold for likely OCD (Saad et al., 2017; i.e., >4). Much of the sample (33.6%) had CBCL‐OCS scores of zero. Clinical interviews using the DISC identified several cases of Attention‐Deficit/Hyperactivity Disorder, Generalized Anxiety Disorder, Depressive Disorders, Oppositional Defiant Disorder, and Conduct Disorder, but no cases of OCD. Full demographic and psychometric data are presented in Table 1. Mean FD across participants was .23 mm (SD = .17), indicating relatively low motion in our sample on average (Power et al., 2012). CBCL‐OCS scores were not correlated with mean FD, *r*(1 1 1) = .13, *p* = .17, nor with age, *r*(1 1 1) = −.11, *p* = .25.

### 3.2 Go/No‐Go task performance and CBCL‐OCS scores

On No‐Go trials, average accuracy was 64.4% (SD = 19.7%), with a mean RT of 438.9 ms (SD = 114.0 ms) for commission errors (failed No‐Go trials). On Go trials, average accuracy was 95.2% (SD = 6.9%) with an average RT of 493.6 ms (SD = 128 ms). Greater accuracy on No‐Go trials (i.e., better inhibitory control) correlated with lower CBCL‐OCS scores, *r*(1 1 1) = −.20, *p* = .03. There were no associations of accuracy on Go trials, or RT on either No‐Go or Go trials with CBCL‐OCS scores (*p*s > .1).

### 3.3 fMRI results

#### 3.3.1 Main effect of errors

**FIGURE 1:**
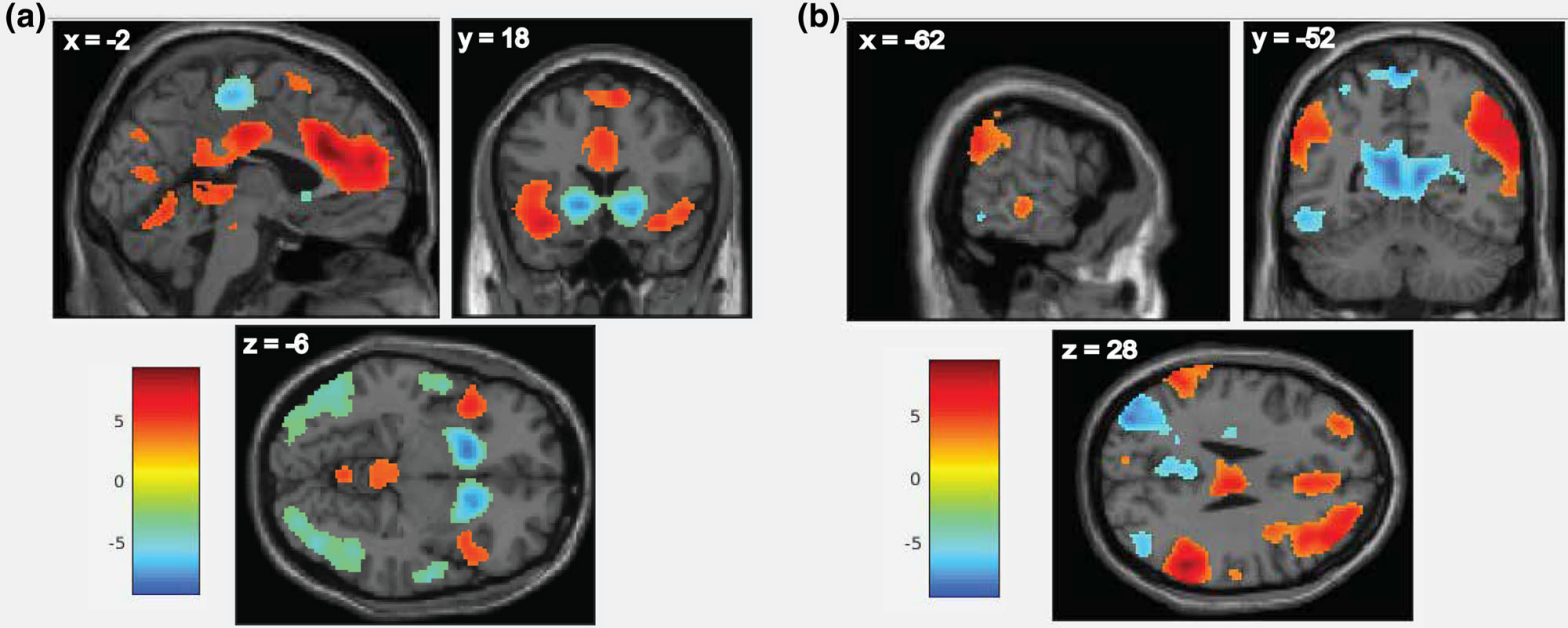
(a) Main effect of error‐related brain activation, with contrast reflecting activity during failed No‐Go trials versus activity during correct No‐Go trials. (b) Main effect of response inhibition brain activation, reflecting activity during correct No‐Go trials versus activity during all Go trials. Data are presented at whole‐brain threshold of *p* < .005, uncorrected. Each color bar displays *t*‐scores, reflecting the relative strength of activation. MNI coordinates of the crosshair intersection are indicated in the top left corner of each brain image.

Error‐processing activated the left anterior, medial and middle cingulate, left inferior frontal gyrus, bilateral insula, and bilateral SMA, and deactivated left putamen, right caudate, a cluster spanning posterior SMA and right post‐central/left paracentral gyrus, bilateral superior temporal gyrus, bilateral inferior occipital gyrus, and left middle occipital gyrus (Figure 1a; Table S2). Results were unchanged when performance regressors were included in the analysis (see “Supplemental Results” in the Supporting Information section).

#### 3.3.2 Primary analysis: neural correlates of CBCL‐OCS scores across whole brain

**FIGURE 2:**
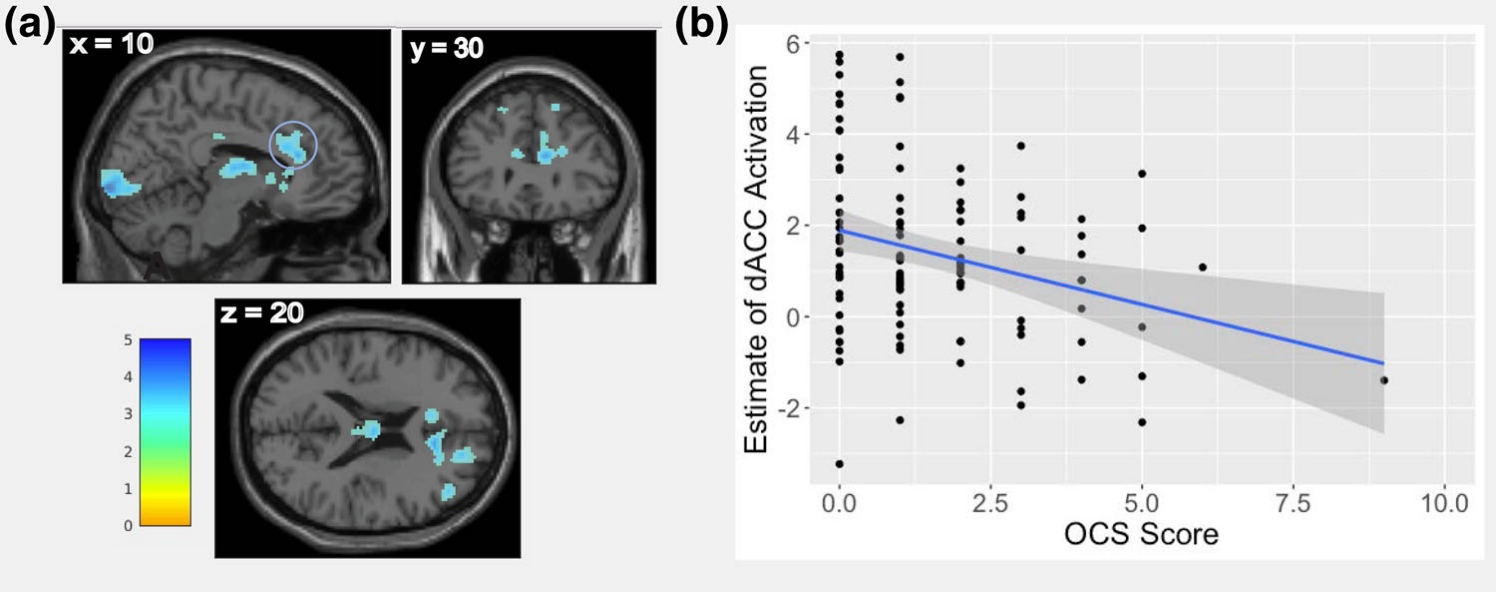
(A) Negative correlation of Child Behavior Checklist obsessive–compulsive subscale score (CBCL‐OCS) with error‐related brain activation, covarying age, FD, and accuracy on No‐Go trials. Data are presented at height threshold of *p* < .005 uncorrected. The color bar displays *t*‐scores, reflecting the relative strength of activation. (B) Scatterplot demonstrating the relationship between CBCL‐OCS and error‐related activation from the dACC cluster (circled in blue in part (A)), covarying age, FD, accuracy on No‐Go trials. The correlation between dACC activation and OCS held when excluding the outlier subject with an OCS score of 9 (*r* = .−29 with outlier vs. *r* = .−26 without outlier, both *p* < .01).

CBCL‐OCS scores were *negatively* correlated with error‐related brain activity in a 485‐voxel cluster in the dACC, as well as clusters in the bilateral putamen, bilateral caudate, left thalamus, and bilateral calcarine/occipital regions (Table 2, Figure 2). By contrast, there were no positive correlations of OCS scores with error‐related activity. Additionally, there were no significant correlations of OCS scores with activity during correct response inhibition. Results remained the same in a regression analysis covarying parental AUD (see “Supplemental Results” in the Supporting Information section). All assumptions for the three regressions tested (on dACC, putamen, and occipital cortex activity) were met.

**TABLE 2:** Error‐related brain activity that was negatively associated with CBCL‐OCS score (failed No‐Go trails vs. correct No‐Go trails, covarying age, FD, and accuracy on No‐Go trials)

Region	Cluster	Coordinates	*Z*‐Value
Right anterior cingulum/anterior cingulate	485	10, 30, 20 20, 32, 22 10, 22, 26	3.64 3.59 3.44
Right thalamus Right putamen Left thalamus Left putamen Right caudate	2169	4, −12, 16 24, 18, −6 −16, −14, 12 −22, 10, 0 14, 0, 12	4.57 4.17 3.76 3.68 3.42
Right calcarine Middle occipital left Right calcarine	1237	12, −96, −4 −38, −92, −2 18, −90, 0	3.95 3.75 3.91

*Note*: There was no error‐related brain activity positively associated with CBCL‐OCS. Region names are based on the region name from the Automated Anatomical Labeling (AAL) atlas. Significant clusters are displayed if *p* < .05, FDR corrected.

#### 3.3.3 Post hoc mediation analyses

**FIGURE 3:**
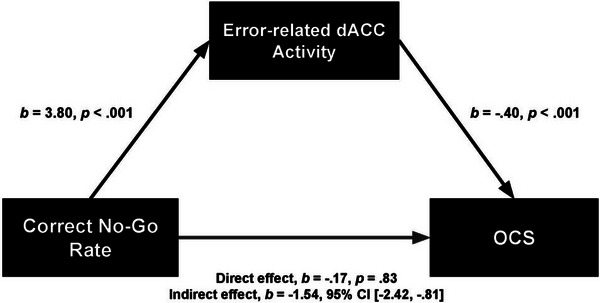
Mediation model reflecting a significant indirect effect of response inhibition performance on obsessive–compulsive symptoms (OCS), through error‐related activity in the dorsal anterior cingulate cortex (dACC). Unstandardized beta values and respective significance, or confidence interval, of each path are indicated on the model.

A post hoc mediation analysis was run to explore whether error‐related activity in the dACC mediated the association between greater inhibitory control (better No‐Go accuracy) and lower OCS. Contrast estimates were extracted from the 485‐voxel dACC region, defined by the effect of OCS scores. A significant indirect effect of correct No‐Go accuracy predicting OCS via error‐related dACC activity, *b* = −1.54, 95% CI [−2.42, −0.81], was observed (Figure 3). That is, greater error‐related dACC activity partially mediated the association between better accuracy on No‐Go trials and lower OCS.

#### 3.3.4 Secondary analyses: specificity of OCS effects on error‐related brain activity

The CBCL‐OCS and CBCL‐Thought problems subscales were correlated at *r* = .67, *p* < .001, and OCS‐ and CBCL‐Anxious/Depressed were correlated at *r* = .80, *p* < .001. Thus, secondary whole brain analyses were conducted to test the effects of CBCL‐Anxious/Depressed and CBCL‐Thought Problems on error‐related activation. Higher CBCL‐Anxious/Depressed scores associated with less activation in the right insula and bilateral calcarine areas of the occipital lobe. There were no other associations of CBCL‐Anxious/Depressed scores, and no associations CBCL‐Thought Problems scores with error‐related brain activity.

A backward linear regression tested the specificity of CBCL‐OCS, relative to all CBCL syndrome subscales, on error‐related activity in the pMFC region defined by the main effect of errors. In addition to lower OCS scores predicting more error‐related pMFC activation (*b* = −.41, *p* < .001), *higher* CBCL‐Withdrawn scores predicted more error‐related pMFC activation (*b* = .19, *p* = .04). Additionally, older age predicted more error‐related pMFC activation (*b* = .15, *p* < .01). No other variables had a significant effect on error‐related pMFC activation in the backward linear regression. The best fitting model included CBCL‐OCS scores, CBCL‐Withdrawn scores and age (*F*(3, 108) = 8.15, *p* < .001) and explained 18.3% of variance.

#### 3.3.5 Response inhibition analyses

For comparison with prior literature implicating deficits of inhibitory control in OCD (Norman et al., 2019), brain activation during response inhibition (correct No‐Go versus correct Go trials) was also examined (Figure 1b; see the Supporting Information section for details). Notably, CBCL‐OCS did not correlate with inhibitory control activation in any region of the brain.

## 4 DISCUSSION

The present fMRI study examined the dimensional relationship of error‐related brain activity to a range of OCS in a community sample of youth. Across levels of OCS, errors engaged the dACC and surrounding regions. With lower OCS, greater error‐related brain activity in dACC was observed. This pattern is consistent with work in similarly aged patients with OCD, in which less severe OCD symptoms were related to greater pMFC‐based error response (Fitzgerald et al., 2018). In addition, lower OCS was associated with greater error‐related activation of putamen, thalamus, and right caudate. Collectively, dACC, putamen, thalamus, and caudate contribute to a CSTC loop that has been implicated previously in OCD (Menzies et al., 2008) and linked to cognitive control functions (Haber, 2016).

### 4.1 Inverse relationship between OCS and dACC activity

The association of greater error‐related dACC activity with less OCS in a community sample of youth extends prior work demonstrating atypical pMFC response to errors in patients with OCD (Carrasco et al., 2013; Norman et al., 2019; Riesel et al., 2019). Consistent with prior literature, a main effect of errors was observed in the dACC and surrounding pMFC, including the SMA and pre‐SMA (Norman et al., 2019; Ridderinkhof et al., 2004). Within the pMFC, the effect of OCS was localized to the dACC. These findings suggest that alteration of dACC‐based mechanisms for processing errors may relate to the expression of OCS, not only in clinically affected individuals (i.e., OCD), but also in those with subclinical OCS.

The functional significance of error‐related brain alterations with OCS remains unclear, and the exact nature of the association may vary with age. Across youth and adults with OCD, meta‐analytic data show excessive pMFC activation to errors (Norman et al., 2016)—a finding that has been widely interpreted to suggest that enhanced brain response to errors may drive obsessive concerns about making mistakes and compulsive attempts at corrective action (Stern et al., 2011). By contrast, in youth with OCD, studies of error‐related brain activity have shown greater (Fitzgerald, Stern, et al., 2010), lesser (Fitzgerald et al., 2013), or no difference (Woolley et al., 2008) in error‐related pMFC activation. Among pediatric patients, a lower severity of OCD symptoms has been found to associate with more pMFC response to errors (Fitzgerald et al., 2018), consistent with the directionality (less OCS, greater dACC activity) reported here. While not directly related to OCS, high overcontrol (i.e., heightened performance monitoring, perfectionism; correlated with anxiety severity) in anxious youth associate with a less error‐related activation of the dACC (Gilbert et al., 2020). These findings are consistent with the inverse relationship between dACC‐based error signaling and OCS reported here. Together, these results support the possibility that greater dACC‐based error signaling may support behavioral adaptation and minimize OCS expression.

### 4.2 CSTC circuitry

In addition to the dACC, youth with less OCS also displayed greater error‐related brain activity in the thalamus, putamen, caudate, and occipital cortex. Dysfunction in these brain areas has been observed consistently with relation to OCD pathology (Menzies et al., 2008), including in pediatric patients (Fitzgerald et al., 2018). The thalamus, caudate, and putamen (in addition to the dACC) are key nodes within CSTC circuitry (Haber, 2016). Broadly, communication among the dACC, thalamus, caudate, and putamen within this circuit helps individuals to attend to motivationally salient stimuli and coordinate goal‐directed behaviors (Peters et al., 2016). In OCD specifically, these regions have been implicated in control and motivational functions (Ahmari & Dougherty, 2015; Fitzgerald et al., 2021). To our knowledge, levels of subclinical OCS have not previously been reported to vary with error‐related function in CSTC circuitry. However, greater subclinical OCS severity has been found to associate with less connectivity of the ventral putamen and medial dorsal thalamus, key CSTC nodes (Suñol et al., 2021). Collectively, these findings suggest that dysfunction within CSTC circuits, previously demonstrated in youth OCD, may extend to youth with subclinical OCS.

There is less evidence evaluating CSTC activity specifically during *errors* in patients with OCD. However, task‐based fMRI findings suggest a dysfunctional response to reward prediction errors in the pMFC and putamen in OCD patients as compared to healthy controls (Hauser et al., 2017). Additionally, there is research supporting dysfunctional activation in pMFC, caudate, thalamus, putamen, and orbitofrontal cortex during response inhibition or conflict trials in patients with OCD as compared to healthy controls (Ahmari & Dougherty, 2015; Marsh et al., 2014). Less activation of these control regions during error‐processing might reflect dysfunction in the mechanism that recruits CSTC for top‐down control after an error (Norman et al., 2019). As we did not hypothesize that CSTC circuitry would be associated with error‐related activity specifically, further investigation of the error‐related behavior of these regions, and their relationship with OCS, is needed.

### 4.3 Brain–behavioral associations

To better understand the association between greater pMFC error‐signaling and less OCS, we tested a mediation model. The mediation was significant: better task performance (greater inhibitory control function) predicted lower OCS by way of increased dACC‐based response to errors. This mechanism aligns with work in healthy individuals, where conflict trials or errors activate the pMFC, thus initiating a feedback loop that involves inhibitory control circuitry to alter behavior (Botvinick et al., 2001). The low‐OCS, healthy youth in this sample who made fewer errors (had more in‐tact inhibitory control abilities), may have also been using a heightened pMFC response to errors to signal effectively to inhibitory control regions, resulting in improved performance after an error. As an adaptive response to errors, engagement of inhibitory control processes might support the reduction of OCS in daily life, resulting in healthy youth with little or no OCS. Secondary analyses tested whether response inhibition activity was related to OCS in our sample, but no relationship was found. Whether this null finding reflects in‐tact inhibitory control behavior across varying levels of OCS is unclear; it is possible that differences in response inhibition brain activity are unobservable until later in life when control circuitry is fully developed.

The finding of more error‐related activity in visual areas of the brain in those with less OCS is consistent with this interpretation. For example, youth with less OCS might be expending more visual resources to attend to errors, subsequently allowing the adaptive recruitment of inhibition–related brain regions during an error. Prior work has identified greater activity in occipital cortex during response inhibition tasks in patients with OCD as compared to healthy controls; this process could reflect an adaptive response where additional resources are used to recruit inhibitory control to preserve task performance (Roth et al., 2007).

### 4.4 OCS specificity

Dysfunctional error‐related pMFC activity may be a general transdiagnostic marker of psychopathology (Riesel et al., 2019). To test whether the error‐related pMFC activity in our sample was related to OCS, as compared to other symptom dimensions, we conducted a backward stepwise linear regression that included all symptom scales of the CBCL. The best fitting regression model found that pMFC activity had the strongest association with OCS as compared to all other symptoms assessed on the CBCL. The only other symptom scale that was correlated with pMFC activity in the final model was the Withdrawn scale. CBCL‐Withdrawn scores were *positively* correlated with pMFC activation, a finding that aligns with a previous work identifying a positive association between ERN amplitude and CBCL‐Withdrawn scores (Hanna et al., 2016).

This specificity was also observed in whole‐brain analyses of the two subscales that make up the CBCL‐OCS score (CBCL‐Thought Problems and CBCL‐Anxious/Depressed); only occipital cortex activity correlated with the CBCL‐Anxious/Depressed subscale (see “Supplemental Results” in the Supporting Information section). Therefore, OCS appears to predict error‐related pMFC activity in our sample over and above other symptoms assessed with the CBCL, demonstrating a specific and inverse relationship between pMFC activity and OCS. The continued study of error‐related pMFC activity in transdiagnostic samples is necessary, though the current analyses provide evidence for activity related to OCS‐specific pathology, thus warranting an OCS‐specific interpretation.

### 4.5 Pattern of inverse OCS/error‐related activity

An inverse correlation between OCS and error‐related brain activity, as observed in the current community sample of mostly healthy youth with OCS, has also been reported in pediatric patients with OCD (Fitzgerald et al., 2018). The convergence of these results should be interpreted carefully, as one finding relates to symptom severity in mostly healthy individuals (with some OCS), and the other to symptom severity in those with clinical OCD. Whether neural correlates of error processing can track OCS dimensionally, across the normal to abnormal spectrum of symptom severity, remains to be seen. Indeed, the inverse relation of error‐related activity and OCS in mostly healthy (i.e., subclinical) *and* clinically severe samples suggests a nonlinear relationship of error‐processing function and OCS across the subclinical–clinical spectrum of severity. Further, interpretations from these studies can be confounded by age and development, which is concurrently changing dimensionally with the brain measure or behavior of interest. To improve inferences that can be made from this inverse relationship, a follow‐up study should examine this relationship at different developmental stages and levels of symptom severity, facilitating understanding of the trajectory of brain‐OCS relations over time. Ultimately, longitudinal study, including youth who progress from subclinical OCS to OCD, will be needed to determine if neural correlates of error‐processing help to quantify risk.

### 4.6 Limitations

Results from the current study provide evidence for error‐related brain activity associated with dimensionally‐measured OCS in a community sample. Future studies may benefit from finding an alternative method for assessing OCS other than the CBCL symptom scale, as the CBCL‐OCS is a parent‐reported assessment of youth OCS. Parent reports of OCS can be unreliable or discordant from self‐reported OCS due to the same associated with obsessions and the fact that symptoms are thought‐based (Rapoport et al., 2000). Additionally, no subjects in the current study met criteria for OCD on the DISC, despite having symptoms that exceed the CBCL‐OCS threshold for OCD. Measuring OCS in young children will continue to be challenging for this reason.

To improve inferences, future studies based on dimensional frameworks such as the Research Domain Criteria could include a normally‐distributed range of symptoms in their sample. In the current sample, a large portion of youth had an OCS score of 0, and none had an OCS score above 9. Moreover, as the OCS symptom scale is a combination of items from two other scales, it does not have a *t*‐score distribution to use in the current analysis. Although these scores were representative of OCS in a community sample (Saad et al., 2017), this distribution does not facilitate the investigation of neural behavior associated with very high OCS scores. Having a larger range of symptom scores in the current study could have provided additional knowledge that is inaccessible in the current analyses.

A final limitation to consider is the cross‐sectional nature of the data analyzed in this paper. We cannot make longitudinal or temporal interpretations of the association between OCS and error‐related brain activity. However, as the MLS is a longitudinal study, once the full data set becomes available, future research *can* assess the longitudinal changes in dACC signaling in this group of participants, in addition to potential worsening of OCS with age. These research investigations will be essential toward understanding whether error signaling is adaptive or pathological, and how its role may change with symptom duration and neural development.

## 5 CONCLUSION

In this community sample youth with OCS, we observed an inverse relationship between OCS and error‐related brain activity, such that youth with lower OCS used more neural resources in dACC, thalamus, putamen, and occipital cortex while making an error during a Go/No‐go task. This finding provides new information about error‐related brain activity in youth, specifically on task‐based neural markers that are associated with subclinical OCS, defined dimensionally. OCS are quite common in nontreatment seeking youth, but prior studies of OCS in youth have indicated that endorsement of OCS at younger ages is predictive of developing OCD or associated comorbidities (Barzilay, 2019; Fullana et al., 2009; Hudziak et al., 2006; Saad et al., 2017). Therefore, the study of neural mechanisms associated with subclinical OCS in youth merits continued investigation.

## CONFLICT OF INTEREST STATEMENT

The authors report no biomedical financial interests or potential conflict of interests. Components of these analyses were presented as an abstract at the Society of Biological Psychiatry's 76th Annual Meeting, April 29–May 1, 2021.

### PEER REVIEW

The peer review history for this article is available at https://publons.com/publon/10.1002/brb3.2941.

## Supporting information

Table S1 Items from the Child Behavior Checklist (CBCL) that make up the OCS score.
Table S2 Main effect of error‐related brain activity (failed No‐Go trails vs. correct No‐Go trails, covarying age, framewise displacement, and accuracy on No‐Go trials).

## Data Availability

The data that support the findings of this study are available from the corresponding author upon reasonable request.
