# Supplementary material for: Error‐related brain activity associated with obsessive–compulsive symptoms in youth
Source: Brain Behav. 2023 Mar 14;13(4):e2941. doi: 10.1002/brb3.2941 (PMC10097091; doi:10.1002/brb3.2941)
Supplement: Supplementary file 1 — Table S1 Items from the Child Behavior Checklist (CBCL) that make up the OCS score. Table S2 Main effect of error‐related brain activity (failed No‐Go trails vs. correct No‐Go trails, covarying age, framewise displacement, and accuracy on No‐Go trials). [file BRB3-13-e2941-s001.docx]

Supplementary Information

**Supplemental Methods**

***Inclusion criteria***

Approximately two-thirds of MLS youth had at least one parent with an alcohol use disorder (AUD), while the remaining third had no parental history of substance use. Exclusion criteria were signs of fetal alcohol syndrome or history of prenatal alcohol exposure; left-handedness; neurologic illness; serious medical illness; treatment with centrally active medications within 6 months; IQ<70; history of psychosis or schizophrenia in a first-degree relative; or MRI contraindications, such as metal implants or claustrophobia (Hardee et al., 2014). For inclusion in error-processing analyses, participants had to be 8–18 years, have usable fMRI data, sufficient error rates for neuroimaging analyses (≥ 5 errors on the fMRI task; Steele et al., 2014), and parent-report data on the Child Behavior Checklist (CBCL; Achenbach, 1999).

***fMRI Acquisition***

Whole-brain blood oxygen level-dependent (BOLD) images were acquired on a 3.0-Tesla General Electric Signa scanner (Milwaukee, WI, USA) using a T2*-weighted single-shot combined spiral in-out sequence (Glover & Law, 2001): repetition time (TR) = 2000 ms; echo time (TE) = 30 ms; flip angle = 90°; field of view (FOV) = 200 mm; 64 × 64 matrix; in-plane resolution = 3.12 mm×3.12 mm; slice thickness = 4 mm. A high-resolution anatomical T1 scan was obtained to perform spatial normalization (three-dimensional spoiled gradient-recalled echo image, TR = 25 ms; min TE; FOV = 25 cm; 256×256 matrix, slice thickness = 1.4 mm). Participants’ motion was minimized using foam pads placed around the head and instructions were provided to participants to keep still during the scans.

***Preprocessing***

Functional images were reconstructed (Noll et al., 2005) and preprocessed using in-house pipelines, including the following steps. Head motion was corrected using FSL 5.0.2.2 MCFLIRT realignment (FMRIB, Oxford, United Kingdom) and runs were excluded if they exceeded 3mm translation or 3-degree rotation in any direction. A total of 6 runs were excluded across three participants. Functional images were slice timing corrected and then spatially normalized to a standard stereotaxic space as defined by the Montreal Neurological Institute using Statistical Parametric Mapping Version 8 (SPM8; Wellcome Trust, London, United Kingdom). A 6mm full-width half-maximum Gaussian spatial smoothing kernel was applied to improve the signal-to-noise ratio and account for anatomical differences between participants.

***First-level analyses***

First-level analyses for each participant were conducted using a general linear model in SPM8, consistent with prior work (Hardee et al., 2014). Regressors for failed No-Go trials (commission errors), correct No-Go trials, and Go trials were convolved with the hemodynamic response function (4000 ms event duration). Motion parameters and white matter signal intensity (derived from an anatomical mask) were also included as regressors, thus removing task-unrelated noise.

***Specificity analyses***

To further test the specificity of error-related pMFC activity with OCS, we examined CBCL-OCS scores relative to all other CBCL-syndrome scores (Anxious/Depressed, Thought Problems, Attention Problems, etc.) as predictors of contrast estimates for error processing extracted from the pMFC. For this analysis, contrast estimates were extracted from an unbiased pMFC region, as defined by the main effect errors across participants.

A dorsal anterior cingulate cortex (dACC) cluster was defined based on the main effect of errors across all participants (see Table S2) unbiased by the relationship to OCS. Use of this independent dACC cluster allowed for the probing of the relationship between dACC activity and other behavioral variables. To further test the specificity of OCS on error-related brain activity, backward linear regression was used to determine which CBCL syndrome scores (OCS, Thought Problems, Anxious/Depressed, Social Problems, Somatic Complaints, Delinquent Behavior, Withdrawn, Attention Problems, and Aggressive Behavior) might predict dACC activity from the error-related contrast estimate, covarying for age, FD, and No-Go accuracy. All variables were entered in a single regression model using Statistical Package for Social Sciences (SPSS), version 28, and a *p*-value threshold of .1 was used to eliminate variables and select the variables included in the final model.

**Supplemental Results**

***Demographic and Clinical Characteristics***

Clinical interviews using the Diagnostic Interview Schedule for Children (DISC) identified several cases of Attention-Deficit/Hyperactivity Disorder, Anxiety Disorders, Depressive Disorders, Oppositional Defiant Disorder, and excessive alcohol use (that did not meet criteria for Alcohol Dependence), but no cases of OCD.

***Response inhibition findings***

During response inhibition, there was greater activation in the bilateral middle temporal gyrus, left inferior parietal lobule, left supramarginal gyrus, and left middle and inferior occipital gyrus. There was deactivation in left posterior cingulate, left middle occipital gyrus, left anterior cingulate, bilateral middle temporal gyrus, inferior temporal lobe, and middle frontal gyrus (Figure 1B).

***Brain activity and OCS without performance covariates***

Additional analyses were run to investigate the effect of Obsessive-Compulsive Symptoms (OCS) on error-related brain activity (failed No-Go trials vs. correct No-Go trials), without co-varying task performance (No-Go accuracy). Age and framewise displacement (FD) were still included as covariates. For these analyses, cluster significance was once again defined by clusters where *p* < .05, with whole brain false discovery rate (FDR) correction. In analyzing brain activity that was negatively correlated with OCS, the results indicated that clusters around the thalamus and occipital cortex met significance. The significant cluster containing the thalamus, also contained the right anterior cingulate cortex (ACC) and the putamen. When the ACC ROI was evaluated for significance on its own (with a 5mm radius), this region only met significance at peak level, *p* = .015, FDR-corrected. At the cluster level, the ACC ROI alone was not significant, *p* = .170, FDR-corrected. There were still no significant *positive* associations between error-related brain activity and OCS.

Similar analyses were run to test the effects of OCS on response inhibition brain activity (No-Go trials vs. Go trials), without co-varying task performance (No-Go accuracy and Go accuracy). Just as with the performance regressors, there were still no significant negative or positive correlations between inhibitory control brain activity and OCS.

***Analyses excluding participants with OCS > 4***

Analyses were run to better understand the effect of participants with OCS scores greater than four, an amount typically representative of full OCD pathology. Seven of the 113 subjects surpassed four OCS. The results held after running the regression analysis for error-related brain activity excluding data from these seven participants.

***Specificity analyses***

As the CBCL-OCS subscale includes items from the CBCL anxiety/depression and thought problems subscales, secondary analyses were run to test the correlation of CBCL-Anxious/Depressed and CBCL-Thought Problems on error-related and response inhibition brain activity. Whole-brain level analyses revealed that the CBCL-Anxious/Depressed subscale was inversely correlated with error-related activity in the occipital cortex and right insula. There were no positive associations between CBCL-Anxious/Depressed and error-related brain activity. There was also no response inhibition activity that related to the CBCL-Anxious/Depressed subscale. For the CBCL-Thought Problems subscale, there was no positive or negative relationship with error-related brain activity. However, response inhibition brain activity in the prefrontal cortex, caudate, insula, and temporal lobe was related to CBCL-Thought Problems symptoms, such that higher OCS was associated with greater activity in these regions. Age, performance, and FD were used as covariates in these regression analyses. Also, regions reported here were significant at *p*FDR < .05, at the cluster level.

***Analyses with parental AUD covariate***

An additional regression analysis was run to test whether a parental alcohol use disorder (AUD) for the children included in our sample impacted our main finding of error-related brain activity associated with OCS. Covariates included age, FD, No-Go accuracy, and presence of a parental alcohol use disorder, and clusters were considered significant at *p*FDR < .05. Our findings remained the same; there was a significant negative effect of OCS on brain activity such that greater OCS was associated with less activity in clusters in the thalamus, occipital lobe, and dACC.

***Post-hoc mediation analysis parameters***

A mediation analysis was conducted as a follow-up test to the primary fMRI results of less error-related activity with greater OCS. Specifically, we tested whether error-related activity in an OCS-defined dACC cluster mediated the association between No-Go accuracy and OCS. The “PROCESS” macro, v3.5 beta in *R,* with bias-corrected 95% confidence intervals (n = 10000), was used to test the significance of the mediation model.

**Supplemental Discussion**

***Brain-behavioral associations***

Secondary analyses tested whether response inhibition activity was related to OCS in our sample, but no relationship was found. Whether this null finding reflects in-tact inhibitory control behavior across varying levels of OCS is unclear; it is possible that differences in response inhibition brain activity are unobservable until later in life when control circuitry is fully developed.

***Specificity***

Specificity of the relationship between error-related brain activity and OCS was also observed in whole-brain analyses of the two subscales that make up the CBCL-OCS score (CBCL-Thought Problems and CBCL-Anxious/Depressed); only occipital cortex activity correlated with the CBCL-Anxious/Depressed subscale.

Supplementary Tables

*Supplementary Table 1.* Items from the Child Behavior Checklist (CBCL) that make up the OCS score.

| **CBCL Item (Item Number)** | **CBCL Syndrome of Each Item** |
| --- | --- |
| Can't get his/her mind off certain thoughts; obsessions (9) | TP |
| Feels he/she might think or do something bad (31) | A/D |
| Feels he/she has to be perfect (32) | A/D |
| Feels too guilty (52) | A/D |
| Repeats certain acts over and over; compulsions (66) | TP |
| Strange behavior (84) | TP |
| Strange ideas (85) | TP |
| Worries (112) | A/D |

*Note*: A/D=Anxious/Depressed, TP=Thought Problems. Figure reproduced from Hudziak et al., 2006.

*Supplementary Table 2.* Main effect of error-related brain activity (failed No-Go trails vs. correct No-Go trails, covarying age, framewise displacement, and accuracy on No-Go trials). Clusters reported met significance at *p*FDR < .05.

| Region | Cluster^a^ | Coordinates^b^ | Z-value |
| --- | --- | --- | --- |
| Posterior medial frontal cortex (pMFC)  Left anterior cingulum  Left medial frontal  Left middle cingulum | 14,549 | -2, 28, 24  -4, 52, 18  -2, -18, 34 | 9.25  7.28  6.67 |
| Right supplementary motor area  Left supplementary motor area | 839 | 10, 16, 60  -8, 12, 66  0, 0, 70 | 5.70  4.38  3.13 |
| Left insula  Left inferior frontal gyrus | 1,663 | -36, 18, -14  -40, 14, 4  -32, 30, 6 | 7.00  5.22  3.30 |
| Right insula | 1,032 | 34, 18, -14  48, 16, -8  40, 12, -4 | 5.38  5.09  4.70 |
| Left putamen  Right caudate  Right medial frontal gyrus | 3,198 | -16, 14, -4  18, 18, -6  4, 42, -16 | -9.17  -7.09  -4.73 |
| Left paracentral gyrus  Right supplementary motor area  Right postcentral gyrus | 12,467 | -6, -26, 56  4, -22, 54  36, -34, 52 | -6.65  -6.65  -6.11 |
| Right superior temporal gyrus | 688 | 64, -18, 0  62, -10, -4  50, -24, 6 | -5.83  -5.47  -3.60 |
| Left inferior occipital gyrus  Left inferior temporal gyrus  Left middle occipital gyrus | 2,589 | -48, -78, -12  -52, -66, -8  -42, -64, 0 | -5.56  -5.37  -5.33 |
| Right inferior occipital gyrus  Right middle occipital gyrus  Right middle temporal lobe | 2,299 | 42, -78, -6  30, -88, -10  46, -56, -4 | -5.28  -4.98  -4.86 |
| Left superior temporal gyrus | 967 | -54, 0, -8  -58, -14, -2  -60, -6, -18 | -5.22  -4.50  -3.81 |

Supplemental References

Achenbach, T. M. (1999). The Child Behavior Checklist and related instruments. In *The use of psychological testing for treatment planning and outcomes assessment, 2nd ed.* (pp. 429–466). Lawrence Erlbaum Associates Publishers.

Glover, G. H., & Law, C. S. (2001). Spiral-in/out BOLD fMRI for increased SNR and reduced susceptibility artifacts. *Magnetic Resonance in Medicine*, *46*(3), 515–522. https://doi.org/10.1002/mrm.1222

Hardee, J. E., Weiland, B. J., Nichols, T. E., Welsh, R. C., Soules, M. E., Steinberg, D. B., Zubieta, J.-K., Zucker, R. A., & Heitzeg, M. M. (2014). Development of impulse control circuitry in children of alcoholics. *Biological Psychiatry*, *76*(9), 708–716.

Hudziak, J. J., Althoff, R. R., Stanger, C., Beijsterveldt, C. E. M., Nelson, E. C., Hanna, G. L., Boomsma, D. I., & Todd, R. D. (2006). The Obsessive Compulsive Scale of the Child Behavior Checklist predicts obsessive-compulsive disorder: A receiver operating characteristic curve analysis. *Journal of Child Psychology and Psychiatry*, *47*(2), 160–166. https://doi.org/10.1111/j.1469-7610.2005.01465.x

Noll, D. C., Fessler, J. A., & Sutton, B. P. (2005). Conjugate phase MRI reconstruction with spatially variant sample density correction. *IEEE Transactions on Medical Imaging*, *24*(3), 325–336. https://doi.org/10.1109/TMI.2004.842452

Steele, V. R., Claus, E. D., Aharoni, E., Harenski, C., Calhoun, V. D., Pearlson, G., & Kiehl, K. A. (2014). A large scale (N=102) functional neuroimaging study of error processing in a Go/NoGo task. *Behavioural Brain Research*, *268*, 127–138. https://doi.org/10.1016/j.bbr.2014.04.001
